# Supplementary material for: A Systematic Review and Meta-Analysis on the Real-World Effectiveness of COVID-19 Vaccines against Infection, Symptomatic and Severe COVID-19 Disease Caused by the Omicron Variant (B.1.1.529)
Source: Vaccines (Basel). 2023 Jan 19;11(2):224. doi: 10.3390/vaccines11020224 (PMC9965204; doi:10.3390/vaccines11020224)
Supplement: Supplementary file 1 [file vaccines-11-00224-s001.zip › vaccines-2105801-supplementary.pdf]

Supplementary Material 1. Search Terms

Pubmed search Covid-19 up to 01 August 2022

"Coronavirus" [mh:noexp] OR "COVID-19" [tiab] OR "COVID 19" [tiab] OR "COVID19" [tiab] OR "SARS-CoV-2" [tiab] OR "Novel coronavirus" [tiab] OR "2019-nCOV" [tiab] OR "coronavirus" [tiab] OR "Coronavirus 2019" [tiab] OR "Coronavirus 19" [tiab] OR "COVID 2019" [tiab] OR "2019 ncov"[tiab] OR "Wuhan coronavirus" [tiab] AND ("COVID-19 Vaccines"[Mesh] OR "COVID-19 vaccine"[tiab] OR "mRNA-1273 vaccine" [Supplementary Concept] OR "mRNA-1273 "[tiab] OR "mRNA vaccine"[tiab] OR "mRNA COVID-19 vaccines"[tiab] OR "ChAdOx1 COVID-19 vaccine" [Supplementary Concept] OR "Ad5-nCoV vaccine" [Supplementary Concept] OR "Ad5-nCoV"[tiab] OR "Covid-19 aAPC vaccine" [Supplementary Concept] OR "Ad26.COV2.S vaccine" [Supplementary Concept] OR "Ad26.COV2.S vaccine"[tiab] OR "adenoviral vector vaccine"[tiab] OR "BNT162 vaccine" [Supplementary Concept] OR "BNT162b2"[tiab] OR "BNT162"[tiab] OR "CoronaVac" [tiab] OR "vaccin*"[tiab] OR "Covid-19 aAPC vaccine"[Supplementary Concept] OR "covid 19 vaccin*"[tiab] OR "covid vaccin*"[tiab] OR "sars cov 2 vaccine*"[tiab] OR "moderna vaccine*"[tiab] OR "Spikevax"[tiab] OR "janssen vaccine*"[tiab] OR "pfizer vaccine*"[tiab] OR "biontech vaccine*"[tiab] OR "Comirnaty"[tiab] OR "tozinameran"[tiab] OR "johnson & johnson vaccine*"[tiab] OR "astrazeneca vaccine*"[tiab] OR "oxford vaccine*"[tiab] OR "novavax vaccine*"[tiab] OR "Sputnik-V"[tiab] OR "Gam-COVID-Vac"[tiab] OR "BNT162b2"[tiab] OR "mRNA-1273"[tiab] OR "AZD1222"[tiab] OR "NVX-CoV2373"[tiab] OR "Gamaleya"[tiab] OR "Sinopharm"[tiab] OR "Sinovac"[tiab] OR "BBIBP-CorV"[tiab] OR "CoronaVac"[tiab] OR "PiCoVacc"[tiab] OR "Convidecia"[tiab] OR "Ad5-nCoV"[tiab] OR "Covaxin"[tiab] OR "BBV152"[tiab] OR "ReiThera"[tiab] OR "Sanofi"[Tiab] OR "MRT5500"[Tiab] OR " ZyCoV-D"[Tiab] OR "CVnCoV"[Tiab] OR "INO-4800"[tiab] OR "AG0302-COVID19"[tiab] OR "Vaxzevria"[Tiab] OR "AZD1222"[Tiab] OR "Covishield"[Tiab] OR "AdCOVID"[Tiab] OR "EpiVacCorona"[Tiab] OR "Aurora-CoV"[Tiab] OR "ZF2001"[Tiab] OR "Soberana 2"[tiab] OR "Pasteur vaccine*"[Tiab] OR "Abdala"[Tiab] OR "CoVLP"[Tiab] OR "Corbevax"[Tiab] OR "COVAC"[Tiab] OR "BBIBP-CorV"[Tiab] OR "Turkovac"[Tiab] OR "Vector Institute vaccine*"[Tiab] OR "CanSino"[Tiab] OR "Bharat Biotech vaccine*"[Tiab] OR "Mass Vaccination"[MeSH Terms] OR"Immunization, Secondary"[Mesh] OR booster[Tiab]) AND ("real-world effectiveness"[tiab] OR "effectiveness"[tiab] OR "association"[tiab] OR "impact"[tiab] OR "vaccine impact"[tiab]) NOT ("Controlled Clinical Trial" [Publication Type] OR "Randomized Controlled Trial" [Publication Type]"Clinical Trial, Phase I" [Publication Type] OR "Clinical Trial, Phase II" [Publication Type] OR "Clinical Trial, Phase III" [Publication Type]) NOT ("animals"[mesh] NOT ("animals"[mesh] AND "humans"[mesh]))

Medline ovid covid vaccine 01 August 2022

Ovid MEDLINE(R) and Epub Ahead of Print, In-Process, In-Data-Review & Other Non-Indexed Citations, Daily and Versions(R) <1946 to 2022>

1 exp Coronavirus/

2 exp Coronavirus Infections/

3 exp COVID-19/

4 COVID 19.mp.

5 covid 19.tw.

6 COVID19.tw.

7 exp SARS-CoV-2/

8 Novel coronavirus.mp.

9 2019-nCOV.mp.

10 Coronavirus/

11 Coronavirus 2019.mp.

12 Coronavirus 19.mp.

13 COVID 2019.mp.

14 2019 ncov.mp.

15 Wuhan coronavirus.mp.

16 exp COVID-19 Vaccines/

17 COVID-19 vaccine.mp.

18 COVID-19 Vaccine*.mp.

19 mRNA-1273 vaccine.mp.

20 mRNA vaccine.mp.

21 mRNA COVID-19 vaccines.mp.

22 mRNA COVID-19 vaccine*.mp.

23 ChAdOx1 COVID-19 vaccine.mp.

24 Ad5-nCoV vaccine.mp.

25 Ad5-nCoV.mp.

26 Covid-19 aAPC vaccine.mp.

27 Ad26 COV2 S vaccine.mp.

28 adenoviral vector vaccine.mp.

29 BNT162 vaccine.mp.

30 BNT162b2.mp.

31 BNT162.mp.

32 CoronaVac.mp.

33 vaccin*.mp.

34 Covid-19 aAPC vaccine.mp.

35 covid 19 vaccin*.mp.

36 covid vaccin*.mp.

37 sars cov 2 vaccine*.mp.

38 moderna vaccine*.mp.

39 Spikevax.mp.

40 janssen vaccine*.mp.

41 pfizer vaccine*.mp.

42 biontech vaccine*.mp.

43 Comirnaty.mp.

44 Comirnaty.mp.

45 tozinameran.mp.

46 johnson & johnson vaccine*.mp.

47 astrazeneca vaccine*.mp.

48 oxford vaccine*.mp.

49 novavax vaccine*.mp.

50 Sputnik-V.mp.

51 Gam-COVID-Vac.mp.

52 BNT162b2.mp.

53 mRNA-1273.mp.

54 AZD1222.mp.

55 NVX-CoV2373.mp.

56 Gamaleya.mp.

57 Sinopharm.mp.

58 Sinovac.mp.

59 BBIBP-CorV.mp.

60 CoronaVac.mp.

61 PiCoVacc.mp.

62 Convidecia.mp.

63 Ad5-nCoV.mp.

64 Covaxin.mp.

65 BBV152.mp.

66 ReiThera.mp.

67 Sanofi.mp.

68 MRT5500.mp.

69 ZyCoV-D.mp.

70 CVnCoV.mp.

71 INO-4800.mp.

72 AG0302-COVID19.mp.

73 Vaxzevria.mp.

74 AZD1222.mp.

75 Covishield.mp.

76 AdCOVID.mp.

77 EpiVacCorona.mp.

78 Aurora-CoV.mp.

79 ZF2001.mp.

80 Soberana 2.mp.

81 Pasteur vaccine*.mp.

82 Abdala.mp.

83 CoVLP.mp.

84 Corbevax.mp.

85 COVAC.mp.

86 BBIBP-CorV.mp.

87 Turkovac.tw.

88 Vector Institute vaccine*.mp.

89 CanSino.mp.

90	Bharat Biotech vaccine*.mp.
91	exp Mass Vaccination/
92	exp Immunization, Secondary/
93	booster.mp.
94	or/16-93
95	real-world effectiveness.mp.
96	effectiveness.mp.
97	exp Association/
98	impact.mp.
99	vaccine impact.mp.
100	or/95-99
101	Controlled Clinical Trial.pt.
102	Randomized Controlled Trial.pt.
103	Clinical Trial, Phase I.pt.
104	Clinical Trial, Phase II.pt.
105	Clinical Trial, Phase III.pt.
106	or/101-105
107	or/1-15
108	94 and 100 and 107
109	108 not 106
110	109 not (Animals/ not (animals/ and Humans/))
111	exp Coronavirus/
112	exp Coronavirus Infections/
113	exp COVID-19/
114	COVID 19.mp.
115	covid 19.tw.
116	COVID19.tw.
117	exp SARS-CoV-2/
118	Novel coronavirus.mp.
119	2019-nCOV.mp.
120	Coronavirus/

121 Coronavirus 2019.mp.

122 Coronavirus 19.mp.

123 COVID 2019.mp.

124 2019 ncov.mp.

125 Wuhan coronavirus.mp.

126 exp COVID-19 Vaccines/

127 COVID-19 vaccine.mp.

128 COVID-19 Vaccine*.mp.

129 mRNA-1273 vaccine.mp.

130 mRNA vaccine.mp.

131 mRNA COVID-19 vaccines.mp.

132 mRNA COVID-19 vaccine*.mp.

133 ChAdOx1 COVID-19 vaccine.mp.

134 Ad5-nCoV vaccine.mp.

135 Ad5-nCoV.mp.

136 Covid-19 aAPC vaccine.mp.

137 Ad26 COV2 S vaccine.mp.

138 adenoviral vector vaccine.mp.

139 BNT162 vaccine.mp.

140 BNT162b2.mp.

141 BNT162.mp.

142 CoronaVac.mp.

143 vaccin*.mp.

144 Covid-19 aAPC vaccine.mp.

145 covid 19 vaccin*.mp.

146 covid vaccin*.mp.

147 sars cov 2 vaccine*.mp.

148 moderna vaccine*.mp.

149 Spikevax.mp.

150 janssen vaccine*.mp.

151 pfizer vaccine*.mp.

152 biontech vaccine*.mp.

153 Comirnaty.mp.

154 Comirnaty.mp.

155 tozinameran.mp.

156 johnson & johnson vaccine*.mp.

157 astrazeneca vaccine*.mp.

158 oxford vaccine*.mp.

159 novavax vaccine*.mp.

160 Sputnik-V.mp.

161 Gam-COVID-Vac.mp.

162 BNT162b2.mp.

163 mRNA-1273.mp.

164 AZD1222.mp.

165 NVX-CoV2373.mp.

166 Gamaleya.mp.

167 Sinopharm.mp.

168 Sinovac.mp.

169 BBIBP-CorV.mp.

170 CoronaVac.mp.

171 PiCoVacc.mp.

172 Convidecia.mp.

173 Ad5-nCoV.mp.

174 Covaxin.mp.

175 BBV152.mp.

176 ReiThera.mp.

177 Sanofi.mp.

178 MRT5500.mp.

179 ZyCoV-D.mp.

180 CVnCoV.mp.

181 INO-4800.mp.

182 AG0302-COVID19.mp.

183 Vaxzevria.mp.

184 AZD1222.mp.

185 Covishield.mp.

186 AdCOVID.mp.

187 EpiVacCorona.mp.

188 Aurora-CoV.mp.

189 ZF2001.mp.

190 Soberana 2.mp.

191 Pasteur vaccine*.mp.

192 Abdala.mp.

193 CoVLP.mp.

194 Corbevax.mp.

195 COVAC.mp.

196 BBIBP-CorV.mp.

197 Turkovac.tw.

198 Vector Institute vaccine*.mp.

199 CanSino.mp.

200 Bharat Biotech vaccine*.mp.

201 exp Mass Vaccination/

202 exp Immunization, Secondary/

203 booster.mp.

204 or/126-203

205 real-world effectiveness.mp.

206 effectiveness.mp.

207 exp Association/

208 impact.mp.

209 vaccine impact.mp.

210 or/205-209

211 Controlled Clinical Trial.pt.

212 Randomized Controlled Trial.pt.

213 Clinical Trial, Phase I.pt.

214 Clinical Trial, Phase II.pt.

215 Clinical Trial, Phase III.pt.

216 or/211-215

217 or/111-125

218 204 and 210 and 217

219 218 not 216

220 109 not (Animals/ not (animals/ and Humans/))

Search strategy embase 01 August 2022

1 exp Coronavirus/

2 exp Coronavirus Infections/

3 exp COVID-19/

4 COVID 19.mp.

5 covid 19.tw.

6 COVID19.tw.

7 exp SARS-CoV-2/

8 Novel coronavirus.mp.

9 2019-nCOV.mp.

10 Coronavirus/

11 Coronavirus 2019.mp.

12 Coronavirus 19.mp.

13 COVID 2019.mp.

14 2019 ncov.mp.

15 Wuhan coronavirus.mp.

16 or/1-14

17 exp COVID-19 Vaccines/

18 COVID-19 vaccine.mp.

19 COVID-19 Vaccine*.mp.

20 mRNA-1273 vaccine.mp.

21 mRNA vaccine.mp.

22 mRNA COVID-19 vaccines.mp.

23 mRNA COVID-19 vaccine*.mp.

24 ChAdOx1 COVID-19 vaccine.mp.

25 Ad5-nCoV vaccine.mp.

26 Ad5-nCoV.mp.

27 Covid-19 aAPC vaccine.mp.

28 Ad26 COV2 S vaccine.mp.

29 adenoviral vector vaccine.mp.

30 BNT162 vaccine.mp.

31 BNT162b2.mp.

32 BNT162.mp.

33 CoronaVac.mp.

34 vaccin*.mp.

35 Covid-19 aAPC vaccine.mp.

36 covid 19 vaccin*.mp.

37 covid vaccin*.mp.

38 sars cov 2 vaccine*.mp.

39 moderna vaccine*.mp.

40 Spikevax.mp.

41 janssen vaccine*.mp.

42 pfizer vaccine*.mp.

43 biontech vaccine*.mp.

44 Comirnaty.mp.

45 Comirnaty.mp.

46 tozinameran.mp.

47 johnson & johnson vaccine*.mp.

48 astrazeneca vaccine*.mp.

49 oxford vaccine*.mp.

50 novavax vaccine*.mp.

51 Sputnik-V.mp.

52 Gam-COVID-Vac.mp.

53 BNT162b2.mp.

54 mRNA-1273.mp.

55 AZD1222.mp.

56 NVX-CoV2373.mp.

57 Gamaleya.mp.

58 Sinopharm.mp.

59 Sinovac.mp.

60 BBIBP-CorV.mp.

61 CoronaVac.mp.

62 PiCoVacc.mp.

63 Convidecia.mp.

64 Ad5-nCoV.mp.

65 Covaxin.mp.

66 BBV152.mp.

67 ReiThera.mp.

68 Sanofi.mp.

69 MRT5500.mp.

70 ZyCoV-D.mp.

71 CVnCoV.mp.

72 INO-4800.mp.

73 AG0302-COVID19.mp.

74 Vaxzevria.mp.

75 AZD1222.mp.

76 Covishield.mp.

77 AdCOVID.mp.

78 EpiVacCorona.mp.

79 Aurora-CoV.mp.

80 ZF2001.mp.

81 Soberana 2.mp.

82 Pasteur vaccine*.mp.

83 Abdala.mp.

84 CoVLP.mp.

85 Corbevax.mp.

86 COVAC.mp.

87 BBIBP-CorV.mp.

88 Turkovac.tw.

89 Vector Institute vaccine*.mp.

90 CanSino.mp.

91 Bharat Biotech vaccine*.mp.

92 exp Mass Vaccination/

93 exp Immunization, Secondary/

94 booster.mp.

95 or/17-94

96 real-world effectiveness.mp.

97 effectiveness.mp.

98 exp Association/

99 impact.mp.

100 vaccine impact.mp.

101 or/96-100

102 exp phase 1 clinical trial/

103 phase 1 clinical trial.mp.

104 exp phase 2 clinical trial/

105 phase 2 clinical trial.mp.

106 exp phase 3 clinical trial/

107 phase 3 clinical trial.mp.

108 exp clinical trial/

109 exp controlled clinical trial/

110 exp randomized controlled trial/

111 or/102-110

112 16 and 95 and 101

113 112 not 111

114 113 not (Animals/ not (animals/ and Humans/))

Database: EBM Reviews - Cochrane Database of Systematic Reviews <2005 to August 01, 2022>
Search Strategy:

----

1 [exp Coronavirus/]

2 [exp Coronavirus Infections/]

3 [exp COVID-19/]

4 COVID 19.mp.

5 covid 19.tw.

6 COVID19.tw.

7 [exp SARS-CoV-2/]

8 Novel coronavirus.mp.

9 2019-nCOV.mp.

10 [Coronavirus/]

11 Coronavirus 2019.mp.

12 Coronavirus 19.mp.

13 COVID 2019.mp.

14 2019 ncov.mp.

15 Wuhan coronavirus.mp.

16 [exp COVID-19 Vaccines/]

17 COVID-19 vaccine.mp.

18 COVID-19 Vaccine*.mp.

19 mRNA-1273 vaccine.mp.

20 mRNA vaccine.mp.

21 mRNA COVID-19 vaccines.mp.

22 mRNA COVID-19 vaccine*.mp.

23 ChAdOx1 COVID-19 vaccine.mp.

24 Ad5-nCoV vaccine.mp.

25 Ad5-nCoV.mp.

26 Covid-19 aAPC vaccine.mp.

27 Ad26 COV2 S vaccine.mp.

28 adenoviral vector vaccine.mp.

29 BNT162 vaccine.mp.

30 BNT162b2.mp.

31 BNT162.mp.

32 CoronaVac.mp.

33 vaccin*.mp.

34 Covid-19 aAPC vaccine.mp.

35 covid 19 vaccin*.mp.

36 covid vaccin*.mp.

37 sars cov 2 vaccine*.mp.

38 moderna vaccine*.mp.

39 Spikevax.mp.

40 janssen vaccine*.mp.

41 pfizer vaccine*.mp.

42 biontech vaccine*.mp.

43 Comirnaty.mp.

44 Comirnaty.mp.

45 tozinameran.mp.

46 johnson & johnson vaccine*.mp.

47 astrazeneca vaccine*.mp.

48 oxford vaccine*.mp.

49 novavax vaccine*.mp.

50 Sputnik-V.mp.

51 Gam-COVID-Vac.mp.

52 BNT162b2.mp.

53 mRNA-1273.mp.

54 AZD1222.mp.

55 NVX-CoV2373.mp.

56 Gamaleya.mp.

57 Sinopharm.mp.

58 Sinovac.mp.

59 BBIBP-CorV.mp.

60 CoronaVac.mp.

61 PiCoVacc.mp.

62 Convidecia.mp.

63 Ad5-nCoV.mp.

64 Covaxin.mp.

65 BBV152.mp.

66 ReiThera.mp.

67 Sanofi.mp.

68 MRT5500.mp.

69 ZyCoV-D.mp.

70 CVnCoV.mp.

71 INO-4800.mp.

72 AG0302-COVID19.mp.

73 Vaxzevria.mp.

74 AZD1222.mp.

75 Covishield.mp.

76 AdCOVID.mp.

77 EpiVacCorona.mp.

78 Aurora-CoV.mp.

79 ZF2001.mp.

80 Soberana 2.mp.

81 Pasteur vaccine*.mp.

82 Abdala.mp.

83 CoVLP.mp.

84 Corbevax.mp.

85 COVAC.mp.

86 BBIBP-CorV.mp.

87 Turkovac.tw.

88 Vector Institute vaccine*.mp.

89 CanSino.mp.

90 Bharat Biotech vaccine*.mp.

91 [exp Mass Vaccination/]

92 [exp Immunization, Secondary/]

93 booster.mp.

94 or/16-93

95 real-world effectiveness.mp.

96 effectiveness.mp.

97 [exp Association/]

98 impact.mp.

99 vaccine impact.mp.

100 or/95-99

101 Controlled Clinical Trial.pt.

102 Randomized Controlled Trial.pt.

103 Clinical Trial, Phase I.pt.

104 Clinical Trial, Phase II.pt.

105 Clinical Trial, Phase III.pt.

106 or/101-105

107 or/1-15

108 94 and 100 and 107

109 108 not 106

110 [109 not (Animals/ not (animals/ and Humans/))]

****

Supplementary Material 2. Summary of quality assessments using JBI appraisal checklist

Criteria

A. Were subjects suitable for study type
   - Case Study: Were groups comparable outside of presence or absence of disease?
   - Cohort Study: Were groups similar and from the same population?
B. Were groups assigned accordingly based on study type
   - Case Study: Were cases and controls properly matched?
   - Cohort Study: Was exposure measured to equally assign to both exposed and non-exposed groups?
C. Was Exposure/Cases accurately measured in a reliable way?
D. Were confounding factors identified?
E. Were strategies in place to manage confounding factors?
F. Were outcomes measured and reported appropriately?
G. Was exposure/follow up period long enough to be meaningful?
H. Were Measures in Place to account for loss due to follow up/shorter period
I. Was appropriate statistical analysis used?

Y/Yes, N/No, U/Unclear, N/A = Not Applicable

Author	Crit. A	Crit.B	Crit. C	Crit. D	Crit. E	Crit. F	Crit. G	Crit. H	Crit. I
(Adams 2022)	Y	Y	Y	Y	Y	Y	N	N	Y
(Altarawneh 2022)	Y	Y	Y	Y	Y	Y	N	Y	Y
(Buchan 2022a)	Y	N	Y	Y	Y	Y	N	N	Y
(Buchan 2022b)	Y	N	Y	Y	Y	Y	N	N	Y
(Carazo 2022)	Y	Y	Y	Y	Y	Y	Y	Y	Y
(Chemaitelly 2022)	Y	Y	Y	Y	Y	Y	N	N	Y
(Collie 2021)	Y	Y	Y	Y	Y	Y	Y	Y	Y
(Fowlkes 2022)	Y	N	N	Y	Y	Y	Y	Y	Y
(Grewal 2022)	Y	Y	Y	Y	Y	Y	Y	Y	Y
(Hansen 2021)	Y	Y	Y	Y	Y	Y	Y	Y	Y
(Jara 2022)	Y	Y	Y	Y	Y	Y	N	N	Y
(Kim 2022)	N	Y	Y	Y	Y	Y	Y	Y	Y
(Kirsebom 2022)	Y	Y	Y	Y	Y	Y	Y	Y	Y
(Lauring 2022)	Y	Y	Y	Y	Y	Y	N	N	Y
(Lind 2022)	Y	Y	Y	Y	Y	Y	Y	Y	Y
(Montez-Rath et al 2022)	Y	Y	Y	Y	Y	Y	Y	Y	Y
(Powell 2022)	Y	Y	Y	Y	Y	Y	Y	Y	Y
(Spensley 2022)	Y	N	Y	Y	Y	Y	Y	Y	Y
(Suarez 2022)	Y	Y	Y	Y	Y	Y	N	N	Y
(Tartof 2022a)	Y	Y	Y	Y	Y	Y	Y	Y	Y
(Tartof, 2022b)	Y	Y	Y	Y	Y	Y	Y	Y	Y
(Thompson 2022)	Y	Y	Y	Y	Y	Y	N	N	Y
(Tseng 2022)	Y	Y	Y	Y	Y	Y	N	N	Y
(UK Health Security Agency, 2022a)	Y	Y	Y	Y	Y	Y	Y	Y	Y
(UK Health Security Agency, 2022b)	Y	Y	Y	Y	Y	Y	Y	Y	Y
(Willett 2022)	Y	Y	Y	Y	Y	Y	Y	Y	Y
(Yoon 2022)	Y	Y	Y	Y	Y	Y	Y	Y	Y
Young-Xu 2022)	Y	Y	Y	Y	Y	Y	Y	Y	Y

Supplementary Material 3. List of covariates used in final analyses of vaccine effectiveness (VE) estimates from the included primary studies

**Study**	**List of covariates used for matched case-control designs or adjustment in cohort studies**
Adams et al.2022	Admission date (biweekly intervals), age, sex, race and ethnicity, U.S Health and Human Services region of admitting hospital
Altarawneh et al. 2022	Matched in a 1:5 ratio according to sex, 10-year age group, nationality, and calendar week of PCR testing in patients with the alpha, beta, and delta variants
Buchan et al 2022a	Age, sex, public health unit region of residence, comorbidities, influenza vaccination status during the 2019/2020 and/or 2020/2021 influenza seasons, positive test >90 days before index date, week of testing, and neighbourhood-level information on median household income, proportion of the working population employed as non-health essential workers, mean number of persons per dwelling, and proportion of the population who self-identify as a visible minority
Buchan et al 2022b	Age (in 10-year age bands), sex, public health unit region of residence, number of SARS-CoV-2 PCR tests during the 3 months prior to December 14, 2020 (as a proxy for healthcare worker status based on the start date of the provincial COVID-19 vaccine program), past SARS-CoV-2 infection >90 days prior to index date, comorbidities associated with increased risk of severe COVID-19, influenza vaccination status during the 2019/2020 and/or 2020/2021 influenza seasons (as a proxy for health behaviours), and neighbourhood-level information on median household income, proportion of the working population employed as non-health essential workers, mean number of persons per dwelling, and proportion of the population who self-identify as a visible minority
Carazo et al. 2022	Age (18–39, 40–59, and ≥60 years), sex, type of employment (as a proxy for socioeconomic status), facility (associated with infection risk and prioritisation for vaccination), testing indication (as a proxy for disease severity), and epidemiological week (to address vaccine roll-out and potential differential in virus exposure opportunities over time between cases and controls)
Chemaitelly et al.2022	Cases and controls were matched two-to-one by sex, 10-year-age group, nationality, and calendar week of PCR test
Collie et al.2022	Age, sex, previous Covid-19 infection, surveillance week, geographic location, and the number of CDC risk factors.
Fowlkes et al.2022	Sociodemographic characteristics, health information, frequency of social contact, mask use, location, and local virus circulation
Grewal et al.2022	Age, sex, public health unit region of residence, week of test, whether they had tested positive for SARS-CoV-2 longer than 90 days ago, comorbidities, and whether there was an active SARS-CoV-2 outbreak in their long term care facility.
Hansen et al. 2021	Age, sex and geographical region, and calendar time as the underlying time scale.
Jara et al.2022	Age, sex, region of residence, nationality, health insurance

Kim et al.2022	Sex, race/ethnicity, number of clinical encounters during 2019, number of underlying health conditions, and days since the previous infection
Kirsebom et al. 2022	Age (in 5-year bands, then everyone age 90 years or older), sex, index of multiple deprivations (decile), ethnic group, geographic region (NHS region), health and social care worker status, clinical risk group status (only available for those aged 64 years and younger, clinically extremely vulnerable (CEV) group status and severely immunosuppressed status
Lauring et al.2022	Calendar date of admission in biweekly intervals, US Department of Health and Human Services region (10 regions), age, sex, and self-reported race and Hispanic ethnicity
Lind et al.2022	Date of test, age, sex, race/ethnicity, Charlson comorbidity score, number of non-emergent visits in the year prior to the vaccine rollout in Connecticut, insurance status, municipality, and social venerability index (SVI) of residential zip code
Montez-Rath et al. 2022	Age, sex, and prior documented SARS-CoV-2 infection.
Powell et al.2022	Age, sex, index of multiple deprivation (quintile), ethnic group, geographic region (NHS region), period (calendar week of onset), clinical risk group status (a separate flag for those aged over and under 16), clinically extremely vulnerable (if aged 16 and above) and previous positivity.
Spensley et al.2022	Age, sex, ethnicity, cause of kidney failure, previous transplant, immunosuppression at time of vaccine, diabetes, prior SARS-CoV2 infection
Suarez Castillo et al. 2022	Matching was based on age, sex , residence, week of testing and presence of a comorbidity
Tartof et.al 2022a	Age, sex, race/ethnicity, Charlson comorbidity index, body mass index, and prior SARS-CoV-2 infection.
Tartof et.al 2022b	Age (18–49 years, 50–64 years, or ≥65 years), the month of emergency department or hospital admission, sex (male or female), race and ethnicity, body-mass index, Charlson Comorbidity Index, receipt of an influenza vaccine in the year before admission, receipt of a pneumococcal vaccine in the 5 years before admission (to adjust for health-care seeking behaviour), and documentation (PCR or lateral flow test) of previous SARS-CoV-2 infection (ever or never).
Thompson et al.2022	Age, geographic region, calendar time (days from August 26, 2021), and local virus circulation in the community and weighted for inverse propensity to be vaccinated or unvaccinated (calculated separately for each vaccine exposure group).
Tseng et al.2022	History of SARS-CoV-2 molecular test, preventive care, number of outpatient and virtual visits, Charlson comorbidity score, obesity (BMI ≥ 30), frailty index, specimen type, immunocompromised status and history of COVID-19. For the hospitalization models, the core variables included history of SARS-CoV-2 molecular test, preventive care, Charlson comorbidity score, obesity (BMI ≥ 30), immunocompromised status and history of COVID-19.

UKHSA 2022	Age (18 to 89 years in 5-year bands, then everyone ≥90 years), sex, index of multiple deprivation (quintile), race or ethnic group, history of foreign travel, geographic region, period (day of test), health and social care worker status, clinical risk-group status, status of being in a clinically extremely vulnerable group, and previously testing positive. These factors were all considered potential confounders and so were included in all models.
UKHSA/Andrews et al.2022	Age group, sex, ethnicity, self-reported vaccination status, geographical region of residence, and the week in which symptoms began
Willet et al.2022	Age, previous infection status, sex, SIMD quartile, and time since most recent vaccination.
Yoon et al. 2022	Propensity to be vaccinated, influenza vaccination history, daily medication use, local virus circulation, study site and occupation
Young-Xu et al. 2022	Age, race, rurality, Veterans Health Administration (VHA) benefits priority and comorbid conditions (cancer, congestive heart failure, hypertension, immunocompromising conditions, obesity and diabetes).

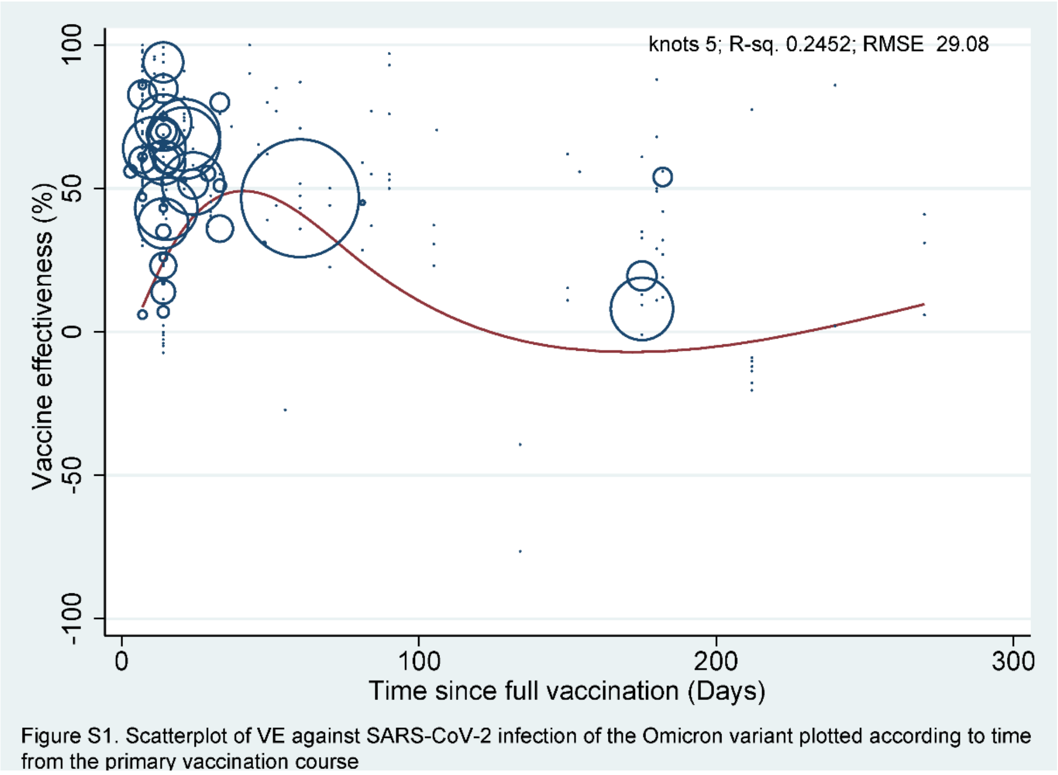

Figure S1. Scatterplot of VE against SARS-CoV-2 infection of the Omicron variant plotted according to time from the primary vaccination course

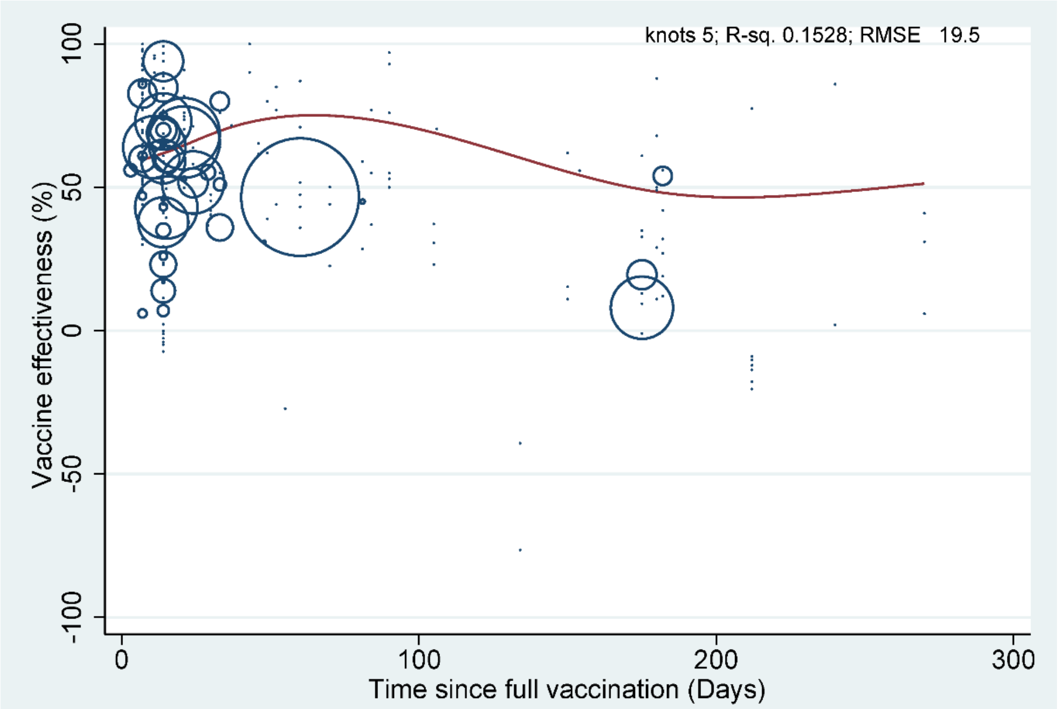

Figure S2. Scatterplot of VE against severe COVID-19 due to Omicron infection plotted according to time from the primary vaccination course

Figure S2. Scatterplot of VE against severe COVID-19 due to Omicron infection plotted according to time from the primary vaccination course

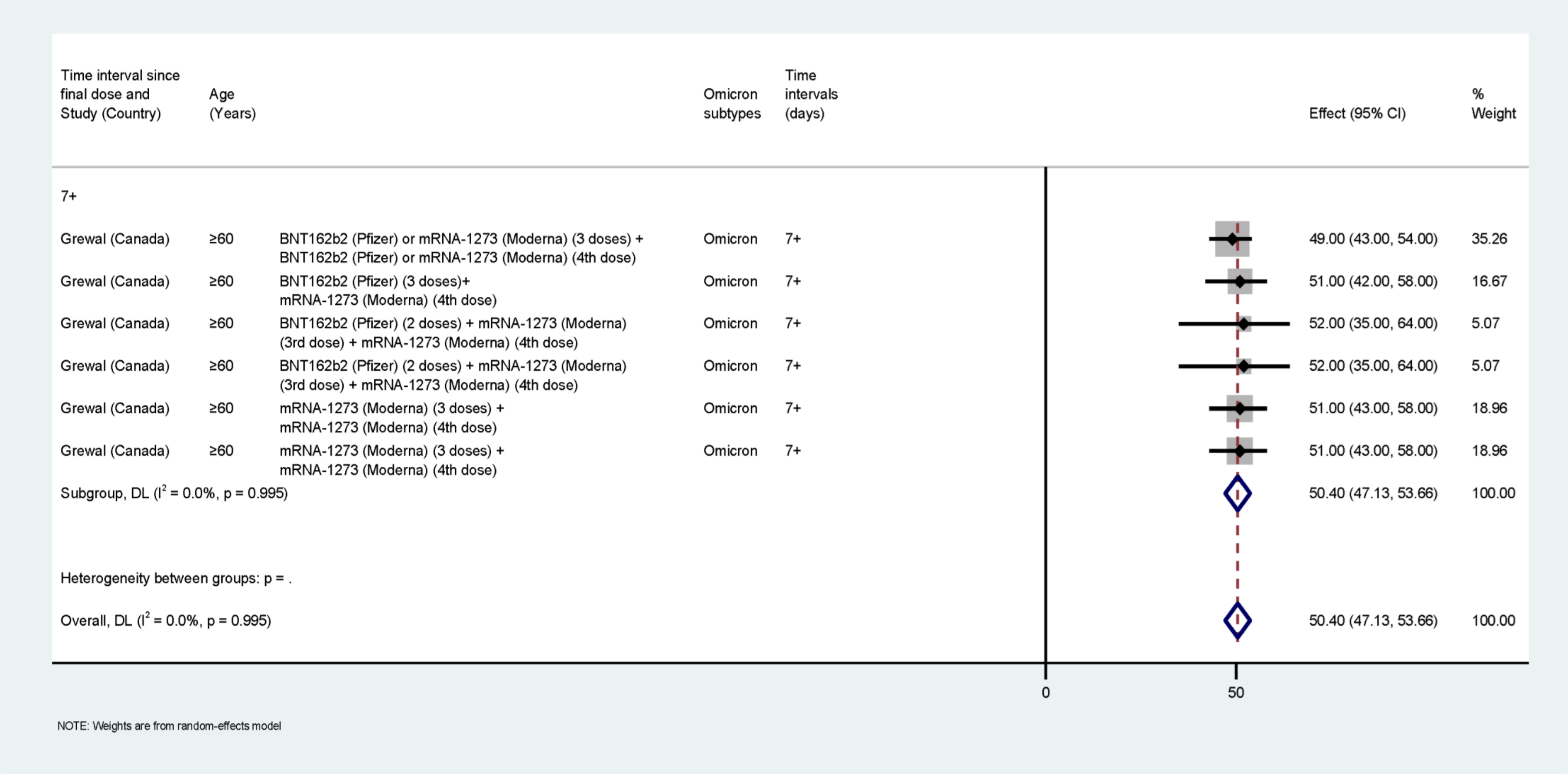

Figure S3. VE estimates against SARS-CoV-2 infection of the Omicron variant after two booster dose in older adults aged ≥ 60 years

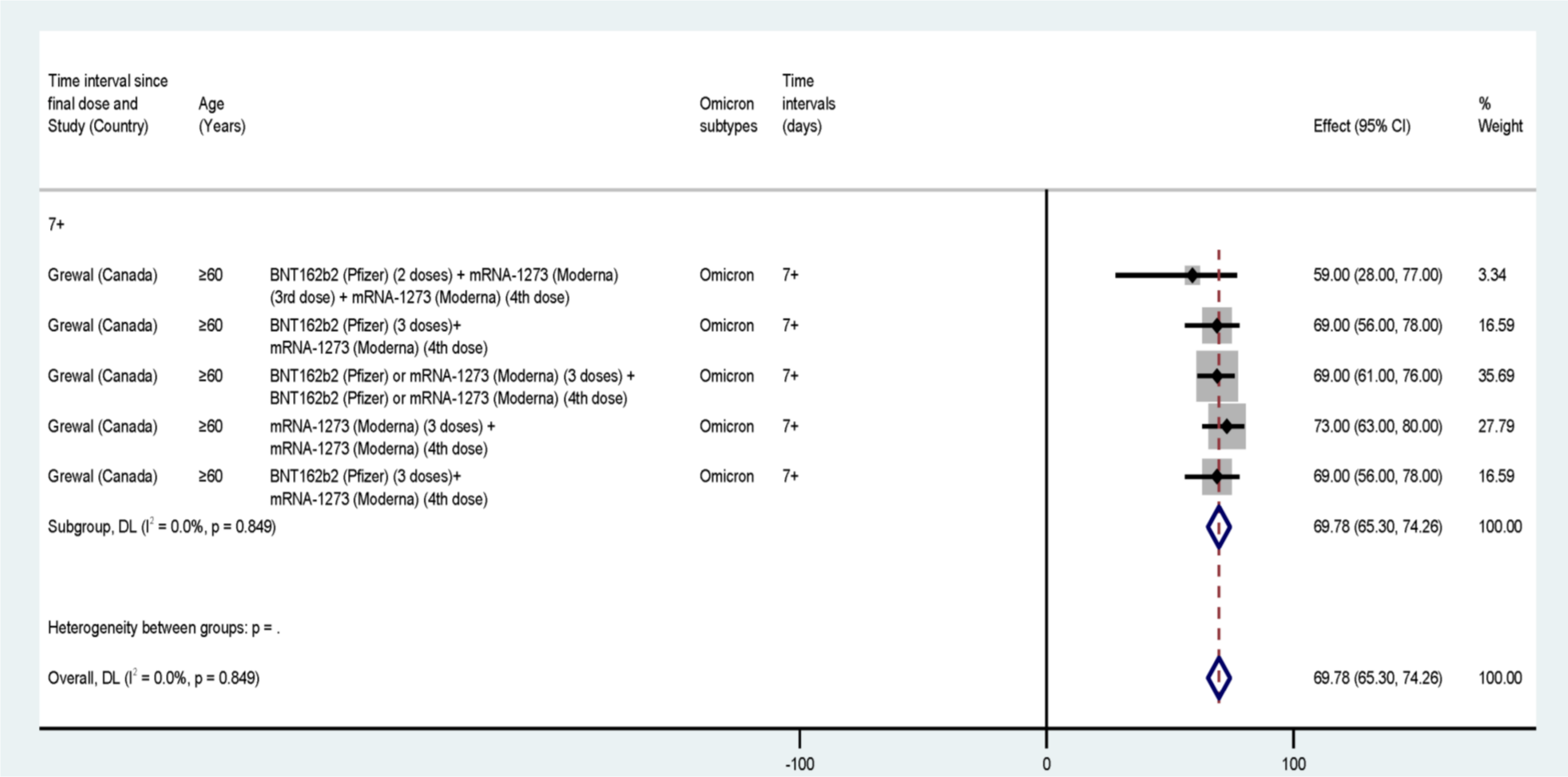

Figure S4. VE estimates against symptomatic Omicron infection after two booster dose in older adults aged ≥ 60 years

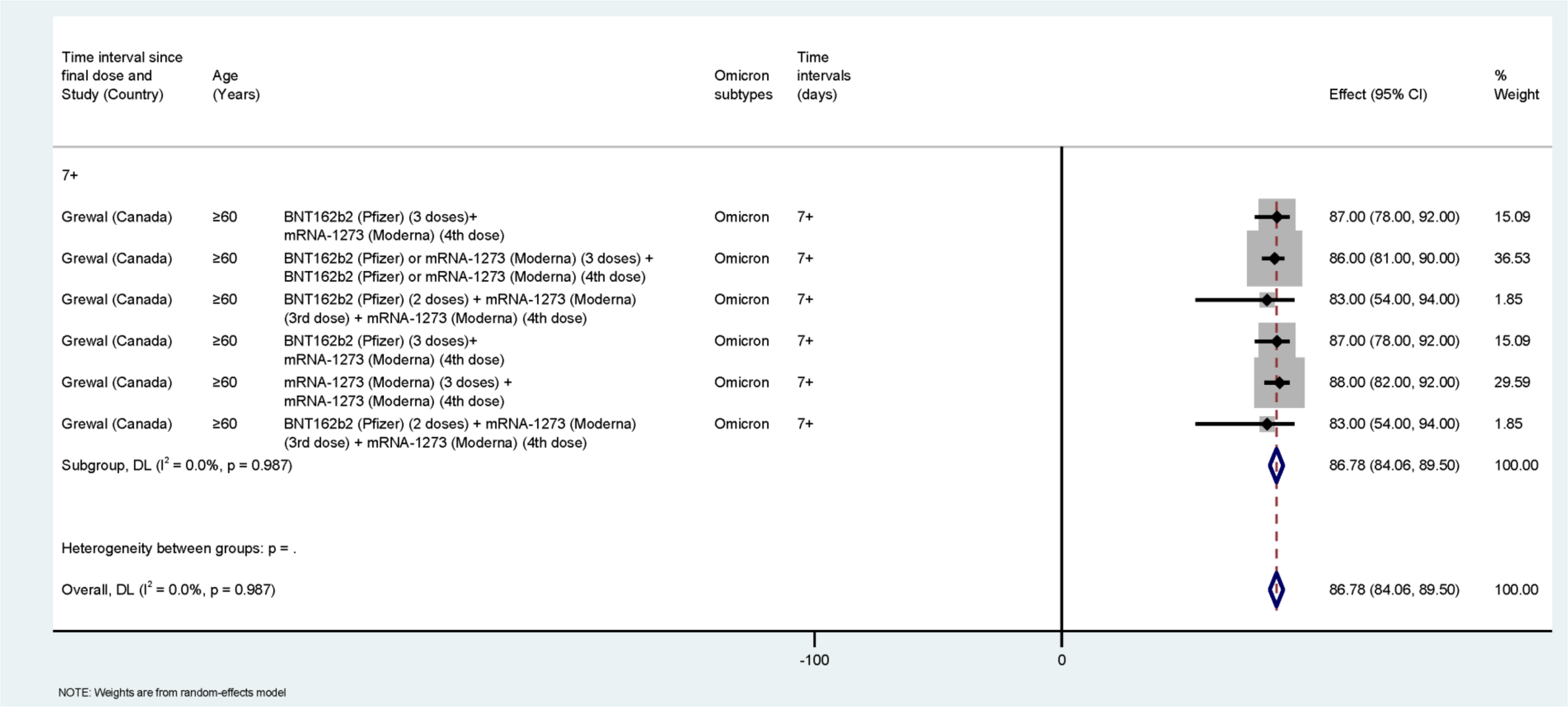

Figure S5. VE estimates against severe COVID-19 due to Omicron infection after two booster dose in older adults aged ≥ 60 years

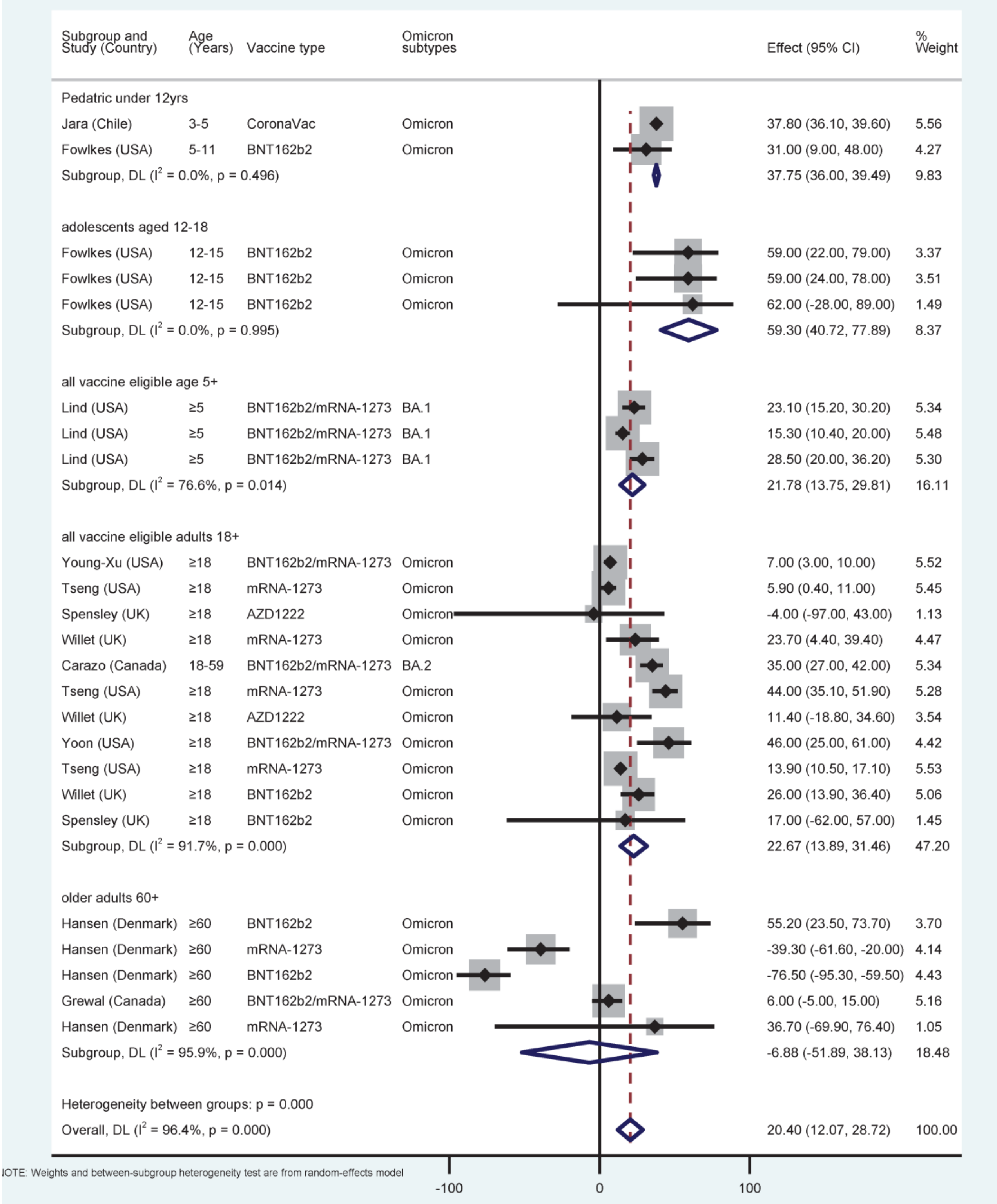

Figure S6. VE estimates against SARS-CoV-2 infection of the Omicron variant after the primary vaccination course, by age group

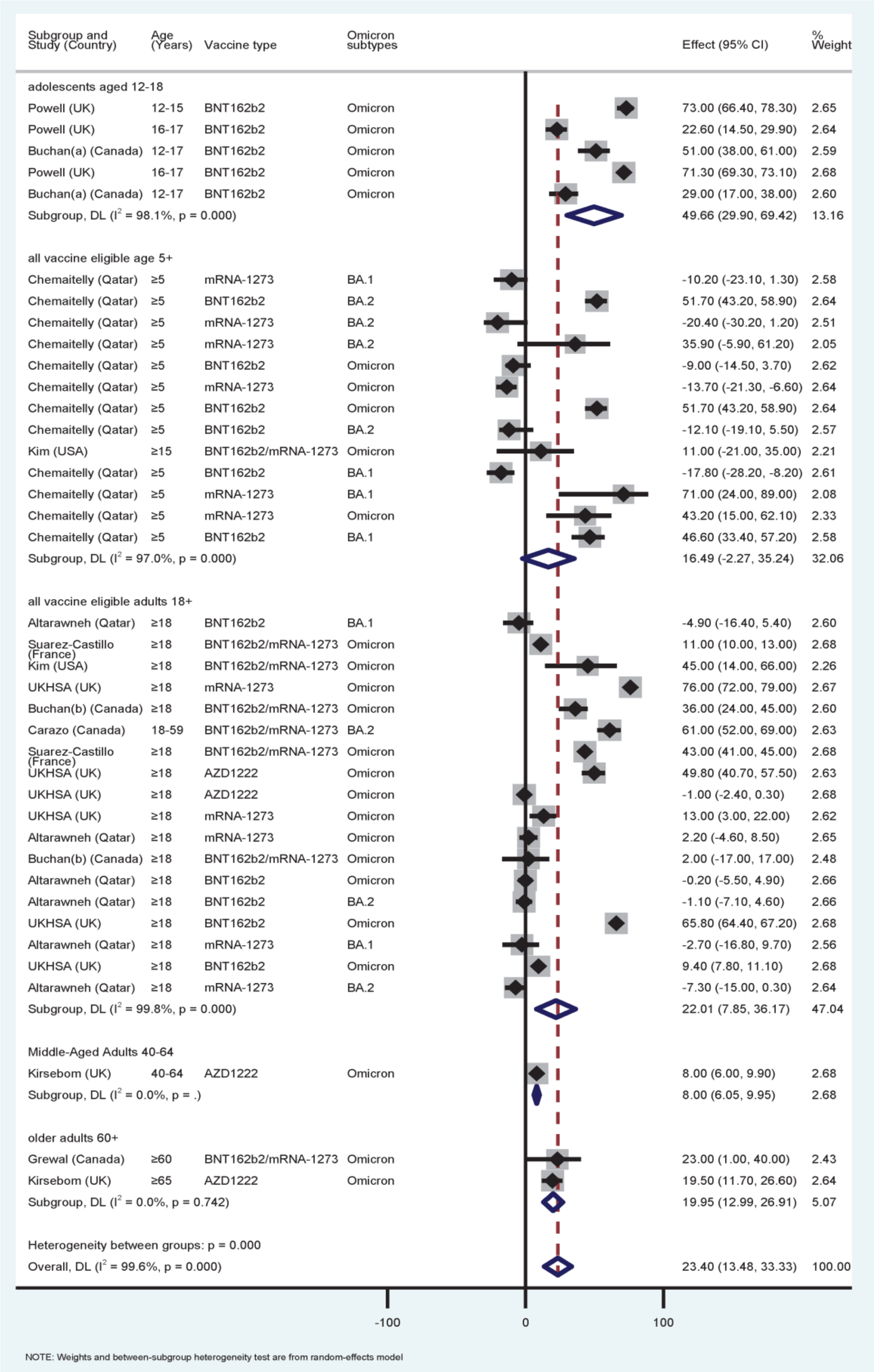

Figure S7. VE estimates against symptomatic Omicron infection after the primary vaccination course, by age group

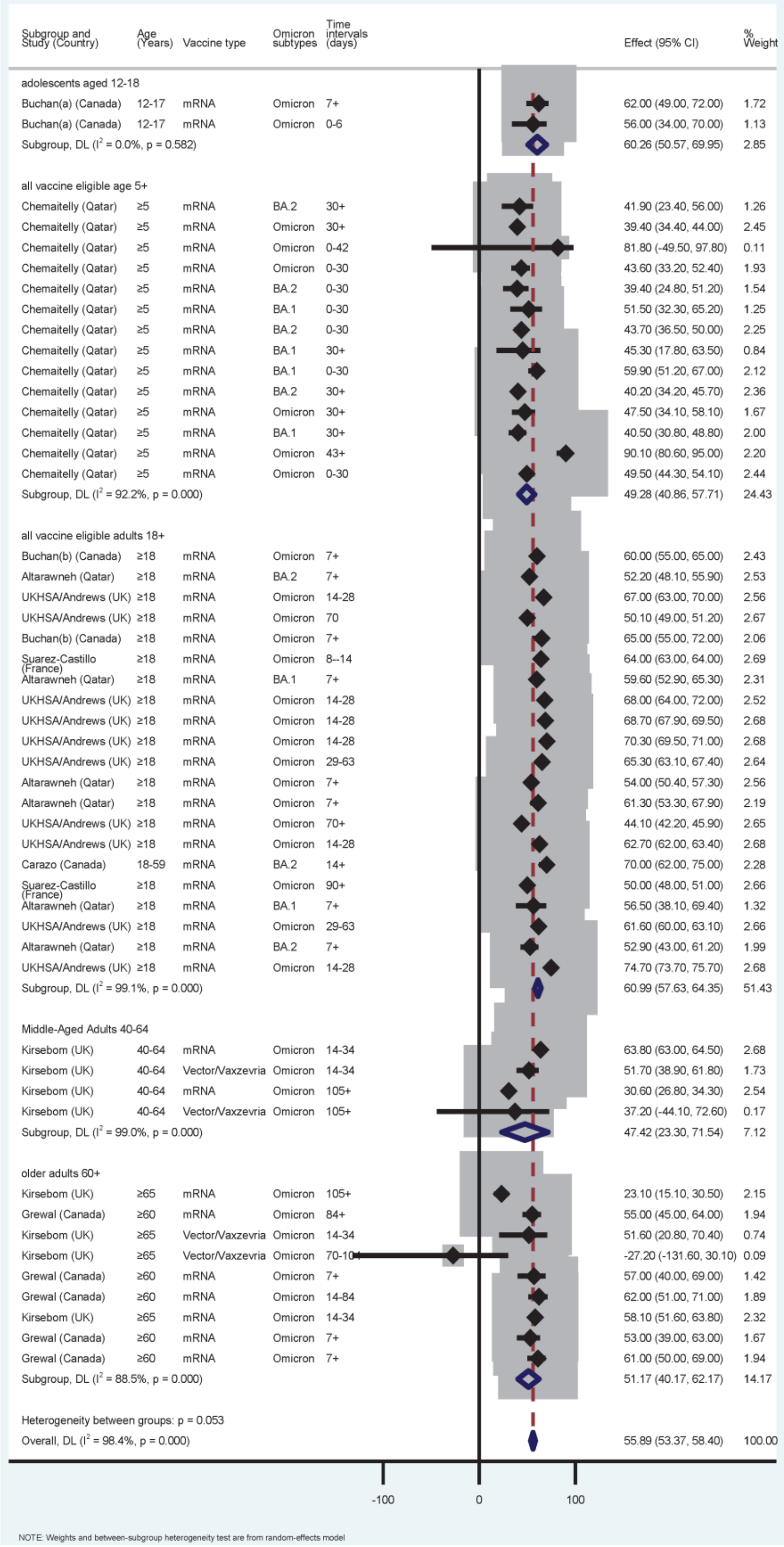

Figure S8. VE estimates against symptomatic Omicron infection after one booster dose, by age group

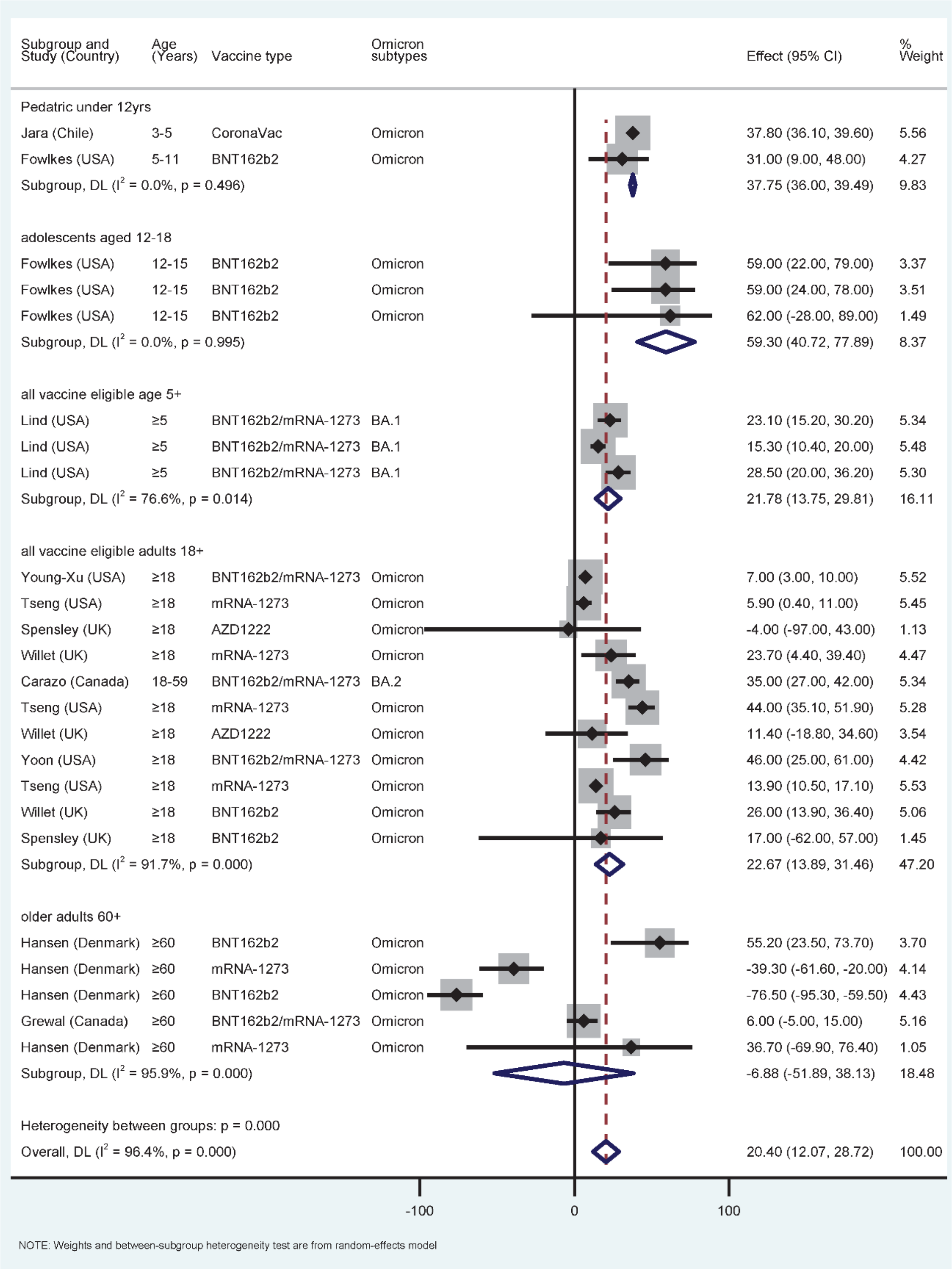

Figure S9. VE estimates against severe COVID-19 due to Omicron infection after the primary vaccination course, by age group

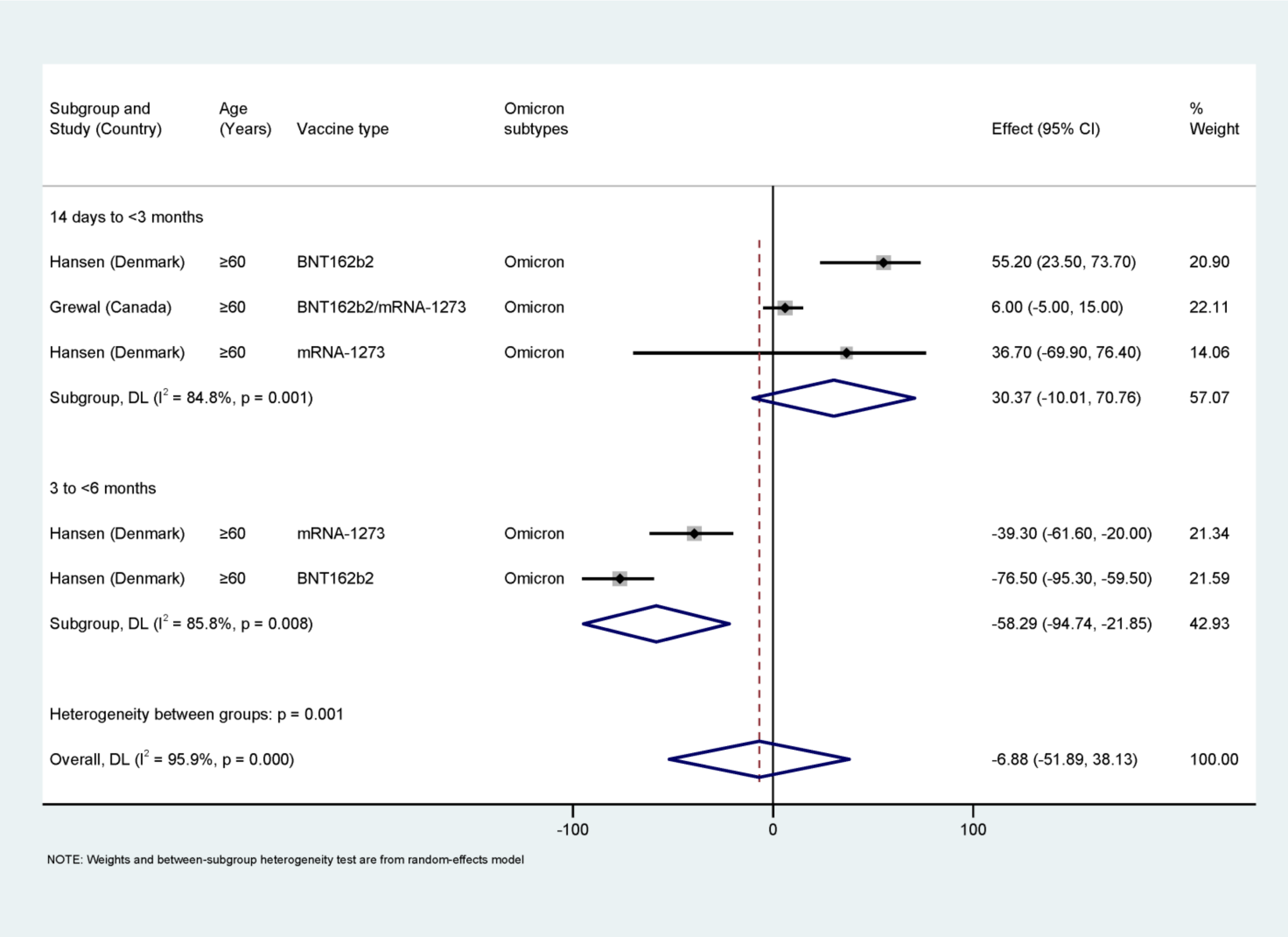

Figure S10. VE estimates against SARS-CoV-2 infection of the Omicron variant after the primary vaccination course, by age group

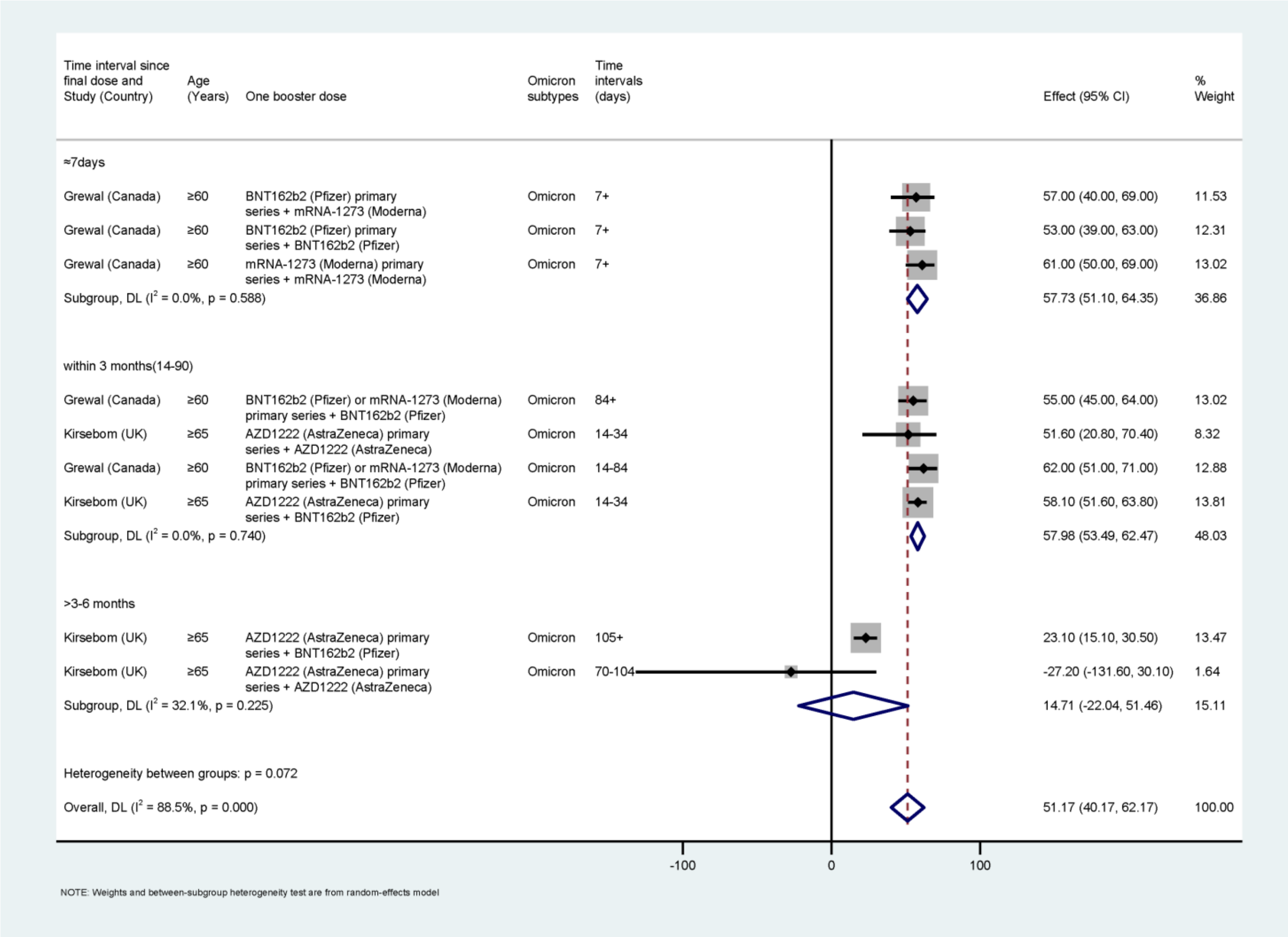

Figure S11. VE estimates against SARS-CoV-2 infection of the Omicron vari-ant after the primary vaccination course in older adults aged ≥ 60 years, by time intervals

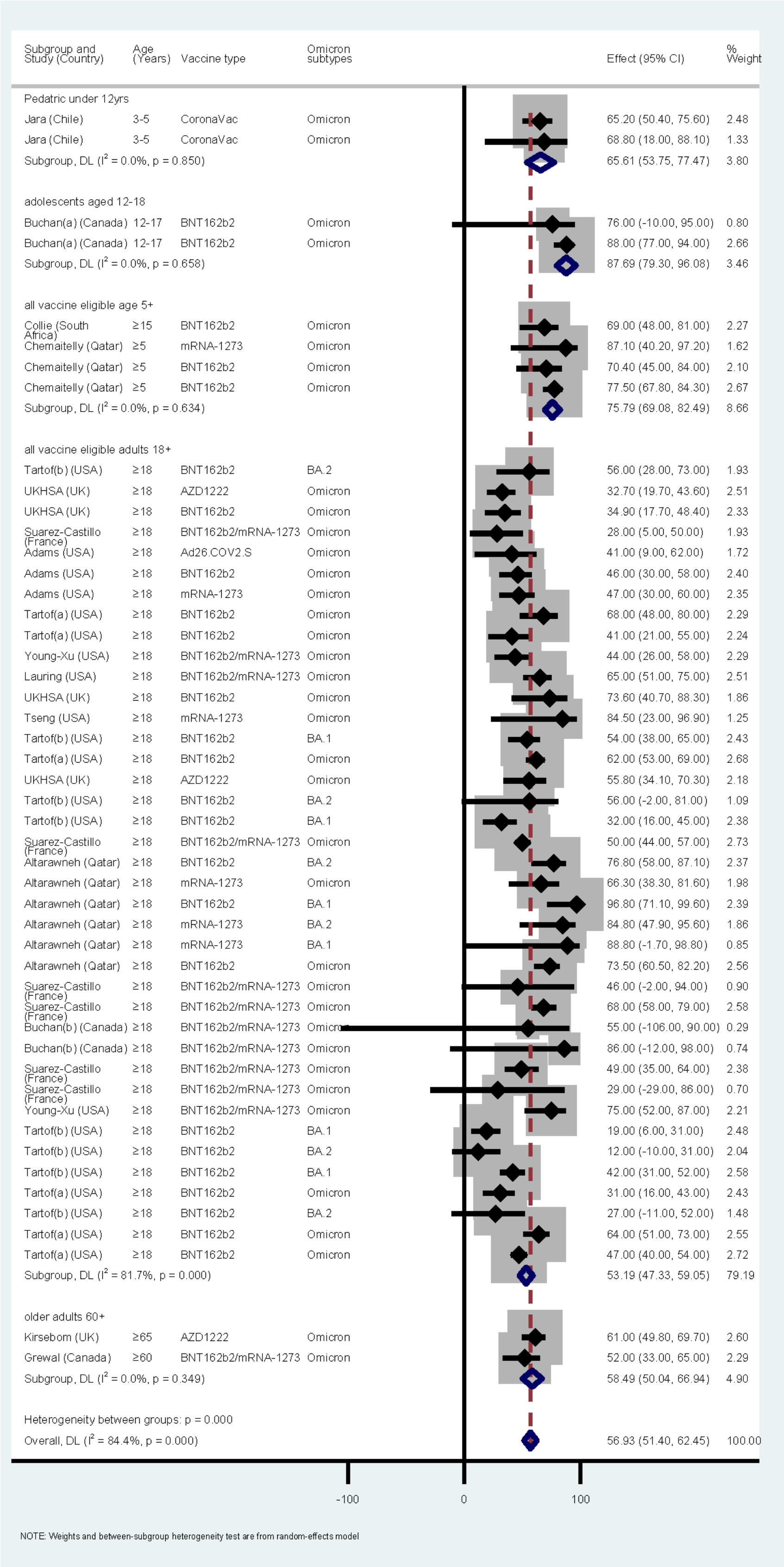

Figure S12. VE estimates against severe COVID-19 due to Omicron infection after the primary vaccination course, by age group

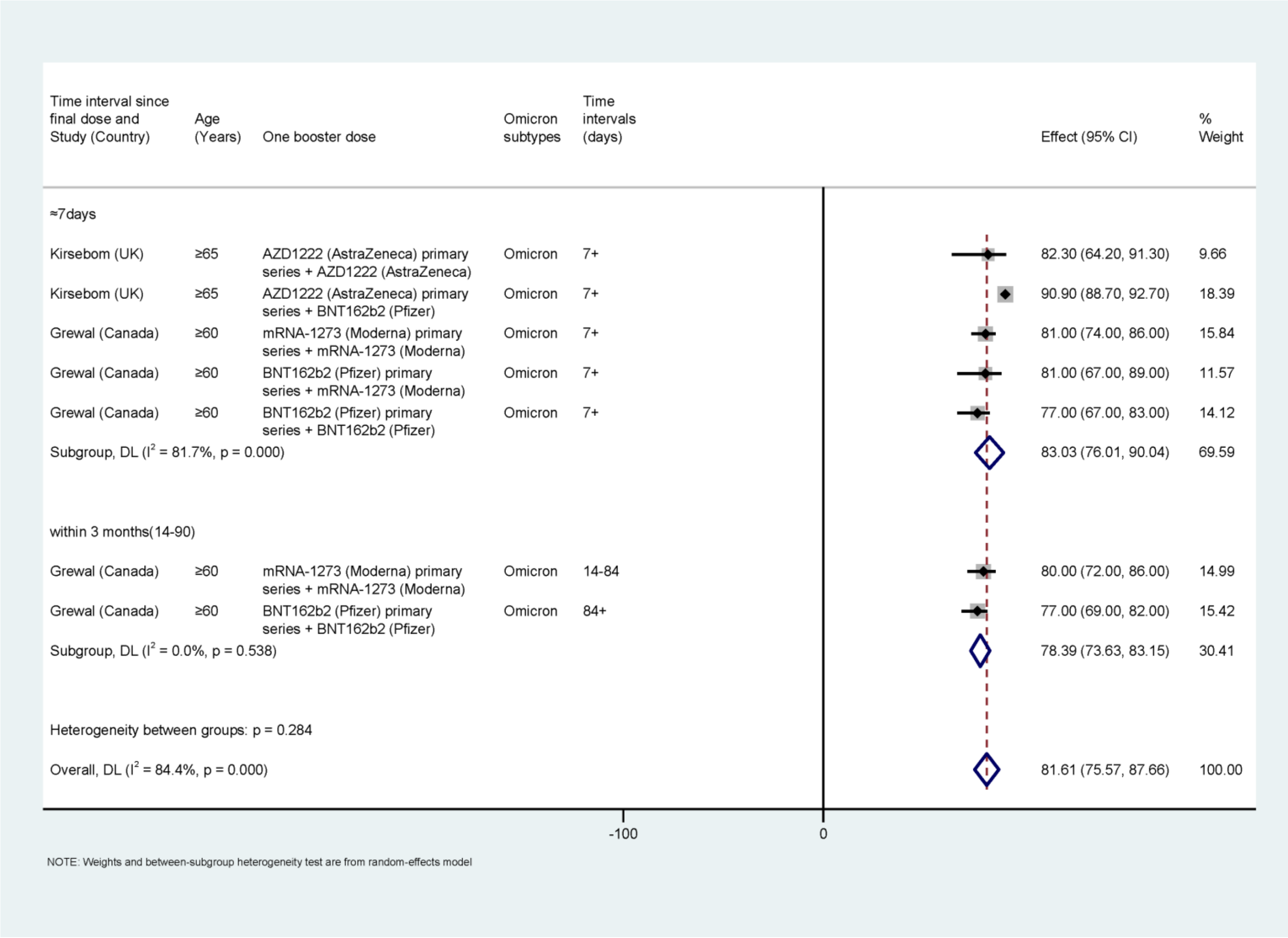

Figure S13. VE estimates severe COVID-19 due to Omicron infection after one booster dose in older adults aged ≥ 60 years, by time intervals

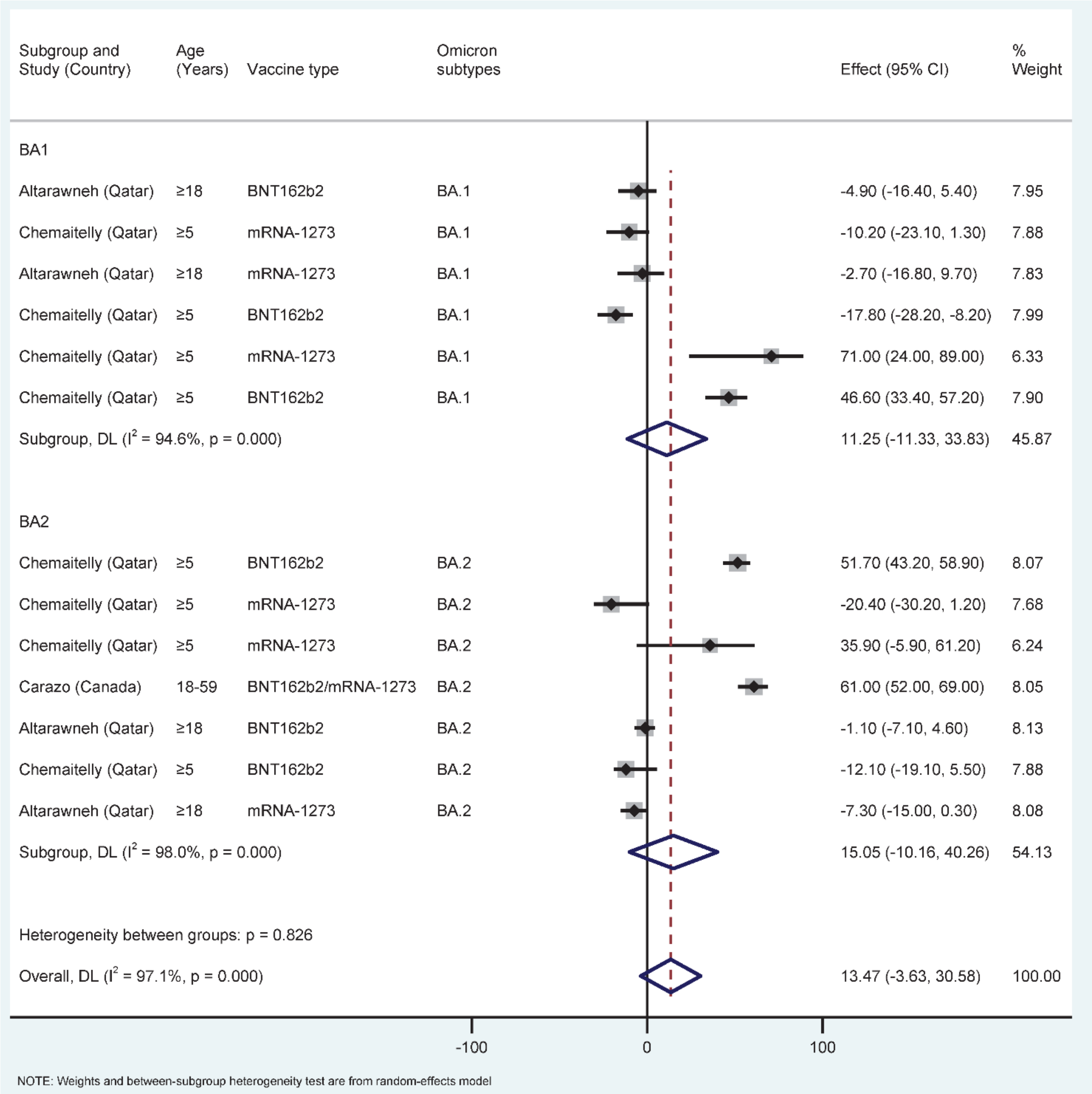

Figure S14. VE estimates against symptomatic Omicron infection after the primary vaccination course, by Omicron sub-variants

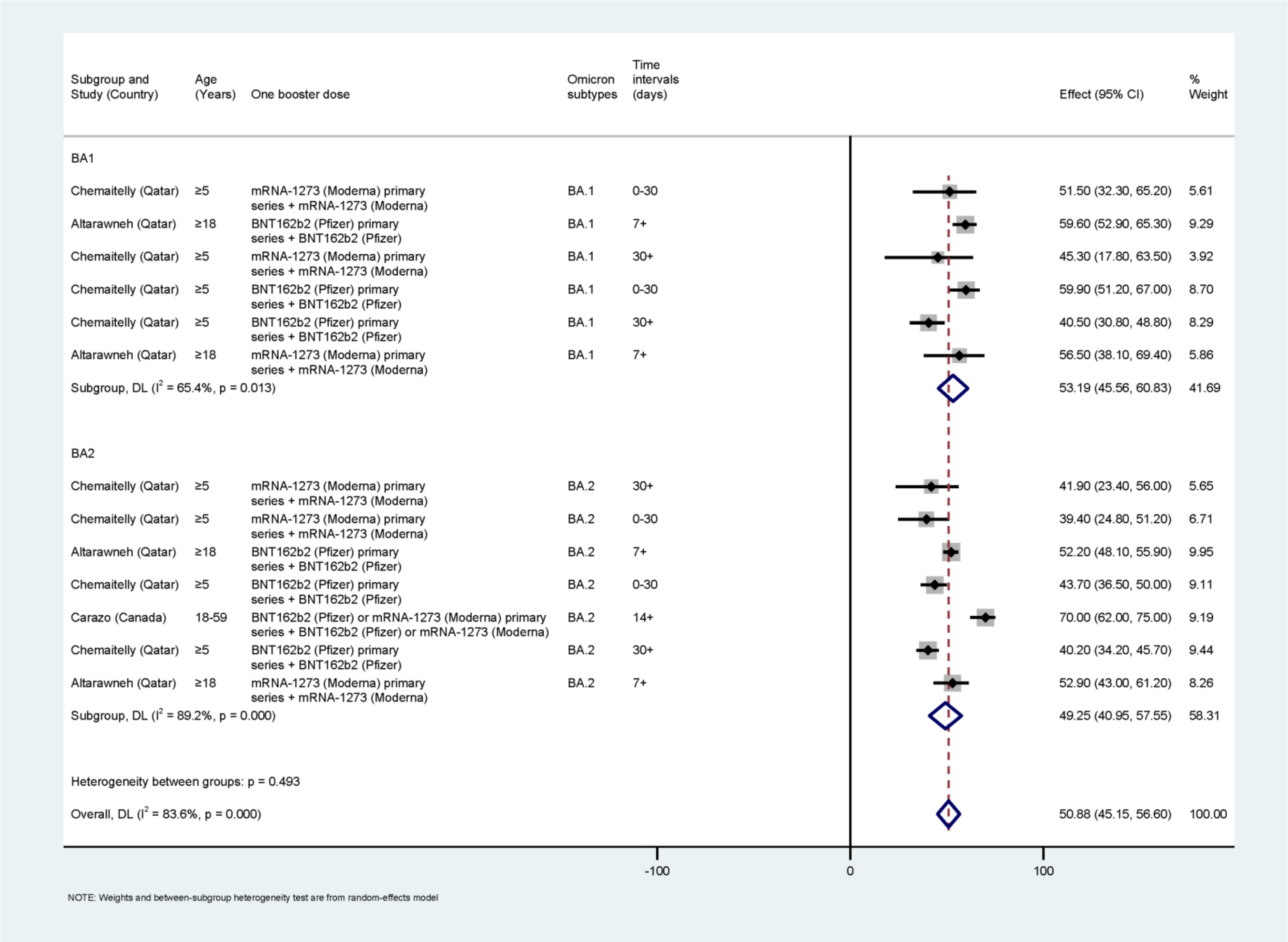

Figure S15. VE estimates against symptomatic Omicron infection after one booster dose, by Omicron sub-variants

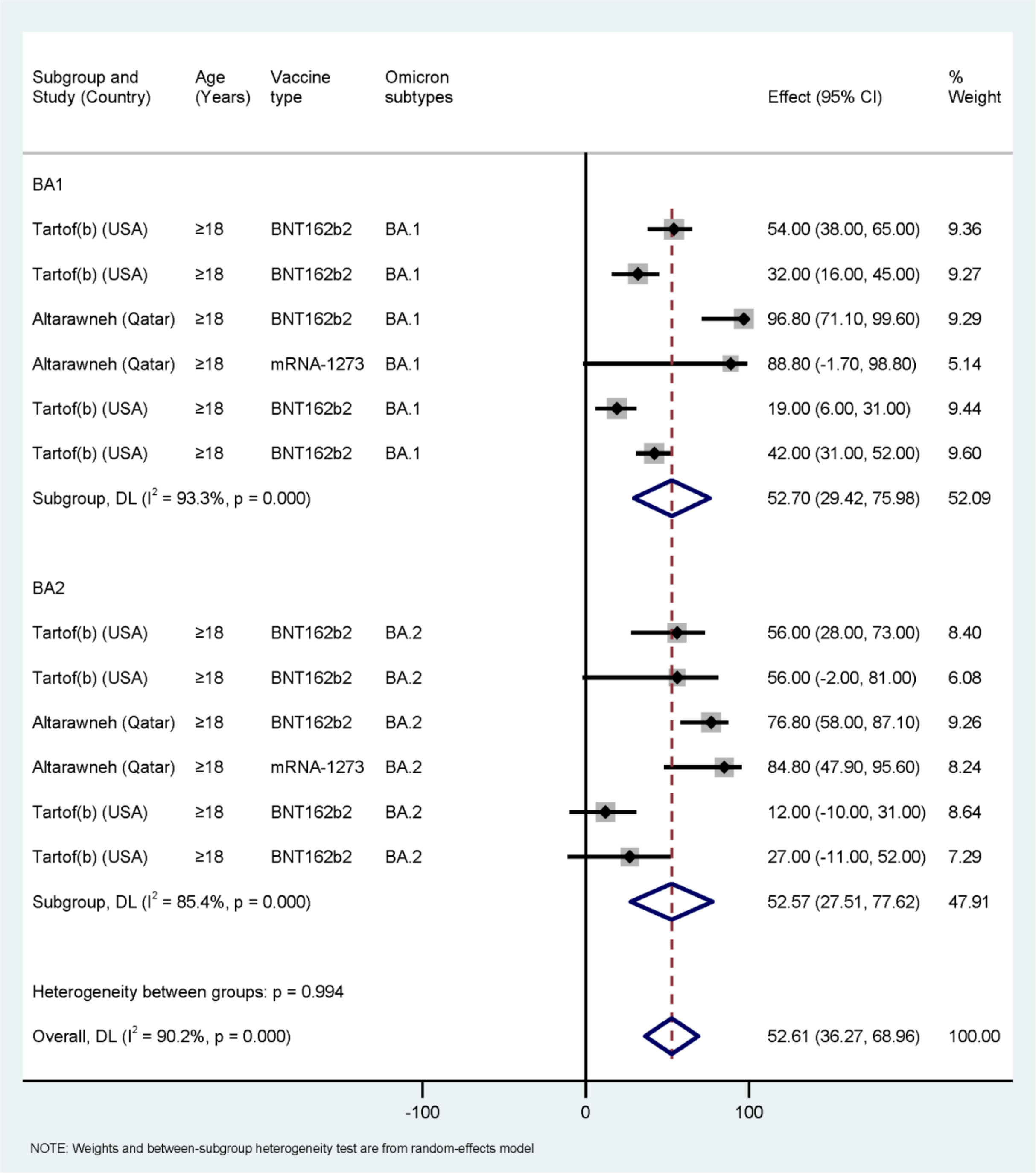

Figure S16. VE estimates against severe Omicron infection after the primary vaccination course, by Omicron sub-variants

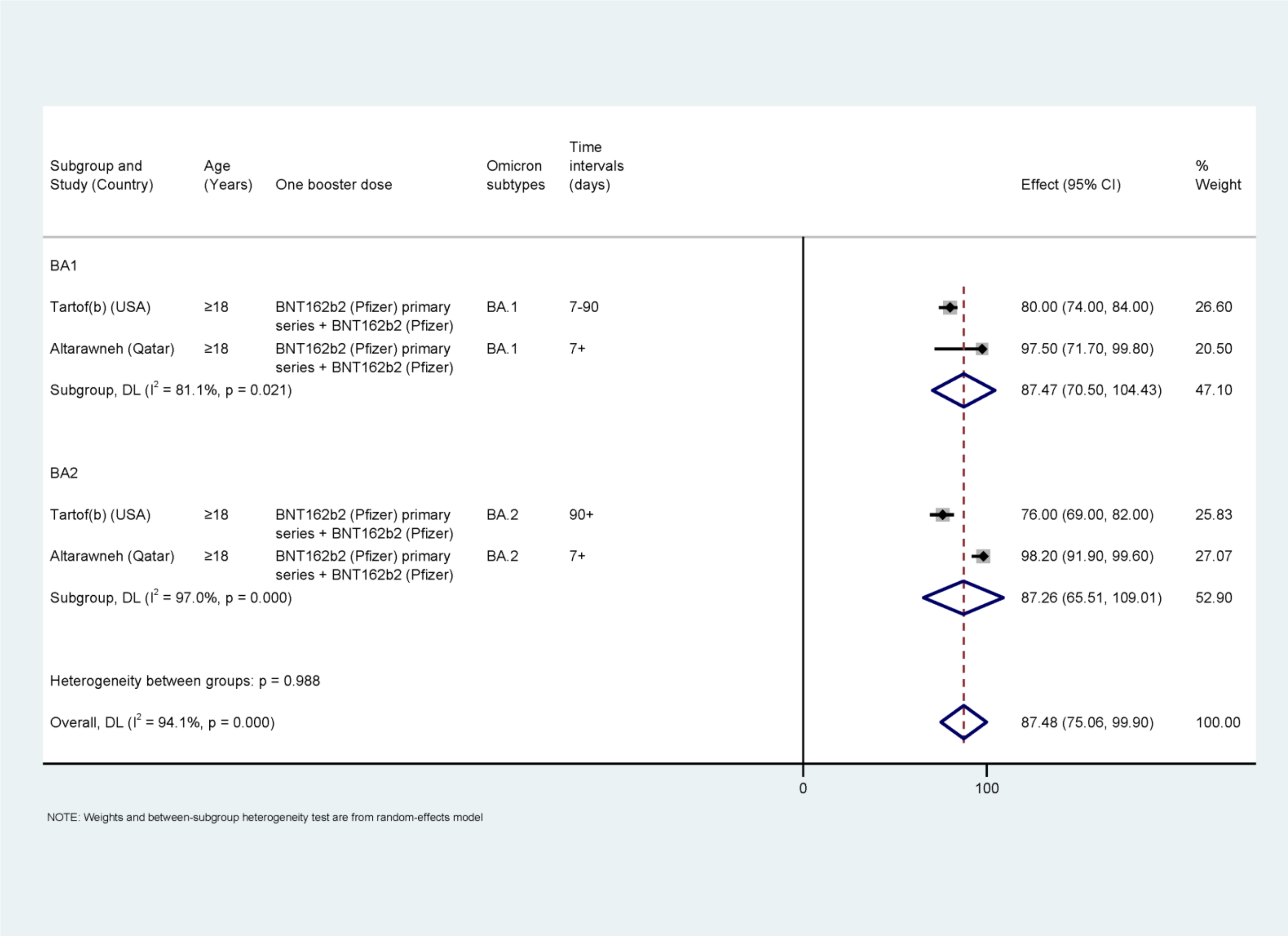

Figure S17. VE estimates against severe Omicron infection after one booster dose, by Omicron sub-variants
